# Categorization in infancy: labeling induces a persisting focus on commonalities

**DOI:** 10.1111/desc.12358

**Published:** 2015-11-05

**Authors:** Nadja Althaus, Kim Plunkett

**Affiliations:** ^1^ Department of Experimental PsychologyUniversity of OxfordUK

## Abstract

Recent studies with infants and adults demonstrate a facilitative role of labels in object categorization. A common interpretation is that labels highlight commonalities between objects. However, direct evidence for such a mechanism is lacking. Using a novel object category with spatially separate features that are either of low or high variability across the stimulus set, we tracked 12‐month‐olds’ attention to object features during learning and at test. Learning occurred in both conditions, but what was learned depended on whether or not labels were heard. A detailed analysis of eye movements revealed that infants in the two conditions employed different object processing strategies. In the silent condition, looking patterns were governed exclusively by the variability of object parts. In the label condition, infants’ categorization performance was linked to their relative attention to commonalities. Moreover, the commonality focus persisted after learning even in the absence of labels. These findings constitute the first experimental evidence that labels induce a persistent focus on commonalities.

## Research highlights

- Infants succeed in forming a new category both in the presence and absence of labels.
- But the processes underlying category formation differ when familiarization objects are presented with or without labels.
- In the absence of labels infants' attention is drawn to variability in category features.
- The presence of labels promotes attention to the commonalities between category exemplars.

## Introduction

It is well established that labels impact the process of visual categorization in both infancy and adulthood: Labels can facilitate category formation (Balaban & Waxman, 1997; Ferry, Hespos & Waxman, 2010; Fulkerson & Waxman, 2007; Lupyan, Rakison & McClelland, 2007; Waxman & Markow, 1995; also see Lupyan, 2012, for a broader account of language modulating cognition), realign category boundaries (Plunkett, Hu & Cohen, 2008), or even impede visual category identification (Robinson & Sloutsky, 2007). Category formation in these studies is typically evaluated in terms of responses to novel stimuli which either belong to the same category as a familiarization set or lie outside the range of this set. In adults, response measures usually involve an explicit judgement about category membership. For example, Lupyan *et al*. (2007) showed that adults learn faster and make fewer errors when learning labeled category exemplars compared to unlabeled exemplars. For infants, the measure is usually a preference for an out‐of‐category novel object over a within‐category novel object. Waxman and Markow (1995) first showed that infants demonstrate a novelty preference for out‐of‐category items over within‐category items when the familiarization set is consistently labelled.

Findings such as these have led some authors to argue that labels act as ‘category markers’ (Yamauchi & Markman, 2000) and that labels ‘act as invitations to form categories’ by ‘highlighting the commonalities’ between objects (Waxman & Markow, 1995), suggesting that during the process of label‐assisted category formation infants (and adults) pay more attention to shared features or feature bundles and, by implication, shift attention away from more variable characteristics of the candidate set of objects. The underlying rationale is that different objects may be represented as members of the same category because shared features are weighted more heavily than dissimilarities. Direct evidence for labels triggering such a mechanism, however, has yet to be obtained.

Much of the debate about the facilitation of categorization in the presence of labels has been centered on the question of whether this effect is language‐specific or due to the presence of an additional acoustic cue (Balaban & Waxman, 1997; Fulkerson & Waxman, 2007; Ferry *et al*., 2010). By 6 months of age, infants’ categorization appears to benefit from speech, but not other types of complex acoustic signal, such as nonhuman primate vocalizations (Ferry, Hespos & Waxman, 2013) – in contrast to 3‐month‐olds, who benefit from nonhuman primate vocalizations, but not other (tone) stimuli (Ferry *et al*., 2010). Despite this growing understanding of how the developing cognitive system becomes specialized to treat speech as a meaningful cue, the mechanism underlying the impact of such cues on category learning remains elusive.

Most infant and adult investigations of the impact of labels on category formation have exploited global measures of category formation, such as error rate or novelty preference, without examining the effect that labeling may have in directing attention to individual object features. Some studies have exploited automatic eye‐tracking to examine category formation in adults and demonstrated selective attention to the diagnostic features for category learning (Rehder & Hoffman, 2005a, 2005b). Althaus and Mareschal (2014) found a transient increase in gaze directed at a low‐variability object part during 12‐month‐olds’ category learning when labels were given, but a direct relationship to categorization performance could not be established. Hence, the hypothesis that labels facilitate categorization by highlighting the commonalities between objects, though plausible, lacks empirical foundation. Conclusive evidence for such a mechanism could be obtained by (a) showing that individual infants’ categorization performance is related to the degree to which they attend to commonalities in the presence of labels (but not in the absence thereof), and (b) showing that a commonality focus persists after learning even when labels are absent.

In this study, automatic eye‐tracking is used to track fine‐grained modulations of attention directed at individual features of objects in an infant categorization task. To examine whether infants learning a category in the presence of labels focus more on commonalities than infants learning about the same category in silence, we constructed a visual category containing two spatially separate features – a leaf and a shell. The spatial separation allowed us to track infants’ attention to each ‘feature’ separately *during learning*. While one of these object parts was of relatively low variability (the leaf), the other (the shell) differed more across exemplars. Familiarizing two groups of 12‐month‐old infants with the same visual material, but providing labels only to one of the groups, we were able to analyse looking patterns with specific reference to commonalities (leaves) vs. the more variable part (shells). By presenting infants with several diagnostic test trials after familiarization, which selectively test for recognition of novelty with regard to the distributional properties of shells and leaves, we were further able to relate individual patterns of looking during familiarization to categorization performance.

## Methods

### Participants

A total of 58 infants participated in this study (mean age: 374 days, range: 355–386 days, 27 girls). Four additional infants were not included in the analysis due to failure to reach the looking time criterion (a minimum of six familiarization trials with recorded looking time). Infants were recruited shortly after birth at the local maternity ward and English was the main language spoken in their home.

### Stimuli

**Figure 1:**
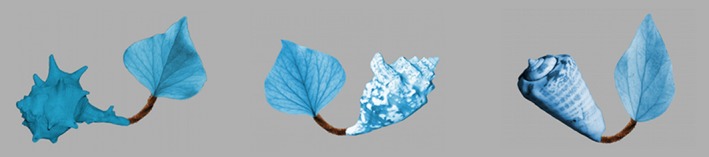
Example familiarization stimuli.

**Figure 2:**
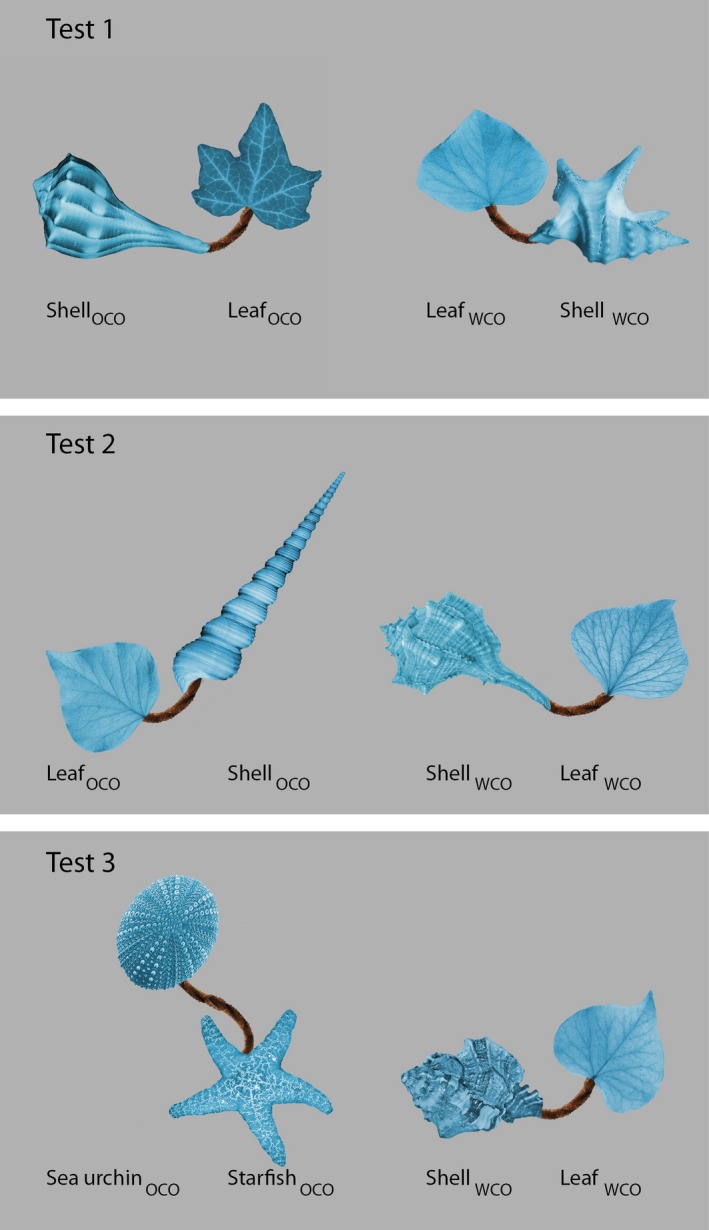
Example Test trials 1, 2 and 3, showing object parts and their role.

A novel category was created by assembling 11 ‘objects’ from images of a shell, a leaf and a pipe‐cleaner (see Figure 1) in the GNU Image Manipulation Program (GNU Image Manipulation Program, 2013). ‘Natural’ objects were chosen as parts for the novel objects in order to reflect the kind of feature variability infants encounter in real life. Photographs of real objects also contain natural shading, providing depth cues that will maximize the impression of a real object. Across the different objects, the leaves were very similar (representing the ‘commonality’ between exemplars), the shells highly variable, and the invariable pipe cleaner served as a connecting limb between these two parts. In order to avoid biases in saliency of individual object parts, all object parts were scaled to contain the same pixel‐volume and colorized to appear blue. Care was taken to make object parts as similar as possible in terms of contrast and perceived hue. For half the objects, the shell was depicted as the left part of the object, and for the other half as the right part. Of these 11 objects, eight served as familiarization stimuli, while the three remaining objects served as familiar (but unseen) objects on three test trials. In addition, three ‘out‐of‐category’ objects were constructed for the test trials (see Figure 2): Test object 1 contained a shell consistent with the category, but an inconsistent type of leaf, Test object 2 contained a leaf consistent with the category but an inconsistent shell, and Test object 3 contained a sea urchin and a starfish instead of a shell and a leaf. On each test trial, the out‐of‐category test object was depicted alongside a ‘familiar’ object, so that four object parts were visible. For convenience, we shall refer to the parts displayed in Tests 1 and 2 as Shell_OCO_, Leaf_OCO_ (together forming the ‘out‐of‐category object’), and Shell_WCO_ and Leaf_WCO_ (together forming the ‘within‐category object’). All images were depicted against a medium grey background on a 40‐inch screen. Objects subtended approximately 14 × 10° visual angle. On the test display, there was a gap of approximately 5° visual angle between out‐of‐category and within‐category objects. In Tests 1 and 2, the two objects were always placed in such a way that the novel part as well as the *corresponding* part of the within‐category object were close to the centre of the screen (e.g. both leaves or both shells were at the centre of the screen, the other parts more peripheral). This permitted direct comparison of looking at these two parts, avoiding biases induced by a more central position of just one of the parts. A recording of the novel label ‘timbo’ (sampling rate 44.1 kHz), pronounced by a female British‐English speaker in an infant‐directed voice, served as the auditory stimulus.

The order of Tests 1, 2 and 3 was maintained constant across infants in order to prevent order effects from obscuring the patterns in the data. Test 1 is designed to evaluate infant sensitivity to leaf variation during familiarization whereas Test 2 evaluates sensitivity to shell variation. As learning is likely to continue beyond the familiarization phase, earlier test trials can impact on infants’ performance on later test trials, making the data harder to interpret (Mather & Plunkett, 2011; Schöner & Thelen, 2006). Since it is easier to learn the feature distribution for a low‐variability part (leaves) than for a high‐variability part (shells), we tested for sensitivity to leaf variability first. The role of Test 3 was to ascertain whether infants were still engaged in the task. A failure to prefer the out‐of‐category object on this test would indicate a lack of engagement, potentially helping to interpret null preferences on earlier trials.

### Procedure

Caregivers were asked to fill in a vocabulary survey (Oxford CDI; Hamilton, Plunkett & Schafer, 2000) prior to their lab visit, which was collected upon their arrival at the lab. After a short warm‐up phase during which written consent was obtained from the caregiver, infants were seated on the caregiver's lap at 75 cm distance from the eye tracker. A 9‐point calibration sequence using the Tobii Studio software was performed up to three times or until all points had been calibrated successfully according to the feedback provided by Tobii Studio.

Half the infants (*N *=* *29) were allocated to the label condition, the other half to the silent condition. Infants were presented with eight familiarization images in pseudo‐randomized order, each for 6000 ms. Four of the familiarization images appeared on the left half of the screen, and four on the right, in no predictable order. Every image was preceded by an attention getter, a small animation at the centre of the screen (with a medium grey background) accompanied by an attractive chiming sound. Animation and sound lasted about 1.5 seconds, with the next trial beginning 2 seconds after the onset of the attention getter. In the label condition, the label ‘timbo’ (duration: 800 ms) was played over a centrally located loudspeaker, 1000 ms after picture onset. Familiarization was followed by three test trials, lasting 10,000 ms each. On the test trials, the three test objects described above were paired with one of the three remaining objects from the familiarization set. Test trials were conducted in silence in both conditions. All stimuli were presented using the Tobii Studio software. Infants’ looking was recorded using a Tobii eye tracker sampling at 120 Hz throughout the familiarization and test phase.

## Results

We first report global measures of looking during familiarization and test (i.e. with respect to whole objects), and then turn to a more detailed analysis of looking directed at individual object parts.

### Analysis

Areas‐of‐interest (AOIs) were defined to contain the area covered by the images of shell and leaf, respectively, plus a 30‐pixel margin around the image outline (corresponding roughly to the eye tracker's 0.5 degree visual angle accuracy). Recorded gaze data were analysed using custom Matlab code.

When reporting looking time at whole objects, we calculate total looking time as the sum of gaze falling on the leaf and the shell. For the purpose of analysing looking directed at individual object parts, we report proportion scores. Since we hypothesized that labels modulate the amount of looking at commonalities (i.e. leaves), we generally report the proportion of Leaf Looking (abbreviated Leaf Looking) for familiarization trials, where just one object is shown at a time. This proportion is obtained for each infant as the duration of gaze directed at the leaf divided by the summed duration of gaze directed at the leaf and the shell. In other words, for familiarization trials the proportions of Leaf Looking and Shell Looking sum to one. On test trials where two objects are shown at a time, we report the proportion of looking directed at the Out‐of‐Category parts (Leaf_OCO_ or Shell_OCO_), which is the proportion of looking time directed at this part divided by looking at all four parts, i.e. the proportion of looking at Leaf_OCO_ is the gaze directed at Leaf_OCO_ divided by the sum of gaze directed at Leaf_OCO_, Shell_OCO_, Leaf_WCO_ and Shell_WCO_. Here, the proportion of looking at Leaf_OCO_ and the proportion of looking at Shell_OCO_ do *not* sum to one and will therefore be reported separately.

### Looking time during familiarization

**Figure 3:**
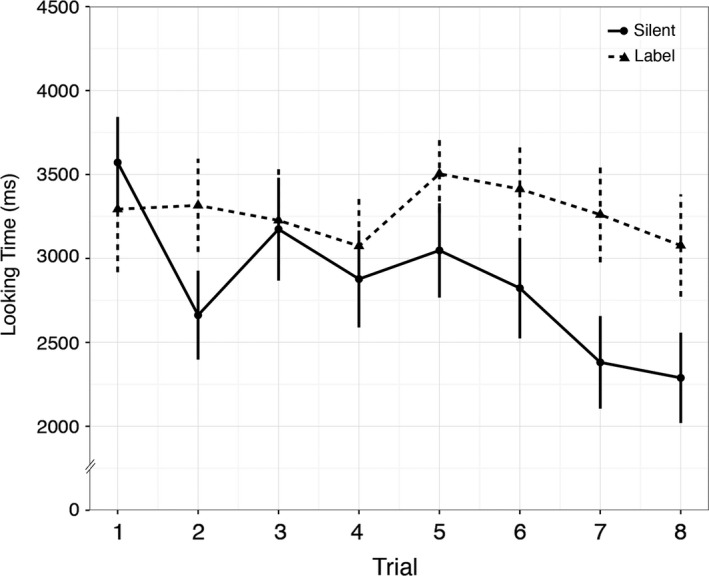
Average looking time across familiarization in the silent and label conditions. Whiskers represent standard errors.

For each infant and each trial, the total looking time was calculated as the sum of fixation time falling on the leaf and shell AOIs. Figure 3 depicts average total looking across infants during familiarization for each trial and condition. Visual inspection suggests a gradual decrease in looking time across trials in the silent condition, whereas looking time is maintained across trials in the label condition. To analyse changes in looking over time, we conducted a mixed effects ANOVA with repeated factor Trial (1 through 8) and between‐subjects factor Condition (Silent, Label). While there was a trend for an effect of Trial (*F*(7, 392) = 1.848, *p *=* *.08), and the linear trend for Trial was significant (*F*(1, 56) = 6.453, *p *=* *.014), this analysis also revealed a significant linear trend for Trial × Condition (*F*(1, 56) = 4.853, *p *=* *.032; all other effects non‐significant, *F*s < 1.3, *p*s* *> .29). This indicates that looking decreased at different rates in the two conditions. Subsequent one‐way repeated measure ANOVAs with factor Trial showed that the linear trend for the silent condition was significant (*F*(1, 28) = 17.673, *p *<* *.001), but not for the label condition (*F*(1, 28) = .042, *p *=* *.84), indicating that infants in the silent condition began to habituate, but infants in the label condition did not. This difference between familiarization in silence and with labels is consistent with previous findings which indicate that auditory stimuli help maintain infants’ attention towards visual stimuli (Baldwin & Markman, 1989; Plunkett *et al*., 2008; Robinson & Sloutsky, 2007).

### Novelty preference at test

Object‐based novelty preference scores were obtained for all test trials by dividing the amount of looking at the out‐of‐category object (i.e. Shell_OCO_ plus Leaf_OCO_) by the total looking time accumulated for the trial (the sum of Shell_OCO_, Leaf_OCO_, Shell_WCO_ and Leaf_WCO_). The results are displayed in Table 1. A mixed‐effects ANOVA with factors Test number (1, 2, and 3) and Condition (Silent, Label) revealed no differences in performance for any of the test trials (all *F*s < .72, *p*s* *> .49).^1^ Planned comparisons against chance (= 0.5, see Table 1) confirmed that infants exhibited a systematic novelty preference (i.e. longer looking at the out‐of‐category object) on all three test trials (although this was only marginally significant for the infants in the silent condition on Test 2).

**Table 1:** Novelty preference scores for Test trials 1, 2 and 3 in both conditions

Condition	Test 1		Test 2		Test 3	
	*M* (*SE*)	*t*	*M* (*SE*)	*t*	*M* (*SE*)	*t*
Silent	.60 (.04)*	2.13	.60 (.05)(*)	1.94	.64 (.05) **	2.72
Label	.61 (.03) ***	4.04	.64 (.04) **	3.51	.65 (.04) **	3.47

Note

Figures marked with (*) are marginally significant (*p *=* *.06), *significantly different from chance at the .05 level, **at the .005 level, ***at the .0005 level.

We then examined which of the two test objects infants first fixated on each test trial. Twenty‐two infants in the silent condition (total *N *=* *29) and 23 in the label condition (total *N *=* *29) first fixated the novel object on Test trial 1 (χ^2^(1, *N *=* *58) = 0, *p *=* *1.0). On Test trial 2, however, the proportion of infants first fixating the novel object differed between conditions. In the silent condition, only seven infants (total *N *=* *27) looked at the novel object first, compared to 21 infants in the label condition (total *N *=* *29; χ^2^(1, *N *=* *56) = 10.3, *p *=* *.001). A further analysis of the difference between longest looks directed at novel vs. familiar objects revealed no effects of condition or test (all *F*s < .868, *p*s* *> .355).

The overall lack of systematic differences between Test trials 1 and 2 in terms of novelty preference scores indicates that infants successfully encoded the distribution of the low‐variability part (leaf) and the high‐variability part (shell), both in the presence and absence of labels, during familiarization. However, the only marginally significant novelty preference score on Test trial 2 for the silent condition points to a less robust category learning process in the absence of labels. This is further underlined by the fact that the majority of infants in the silent condition directed their first look towards the familiar object as they encountered this trial. It seems therefore likely that infants in the silent condition formed a highly accurate, robust representation of the low‐variability part (leaf) but their representation of the high‐variability part (shell) was more fragile.

### Part‐based looking during familiarization

Objects were constructed to have *spatially separate* features, permitting a finer‐grained analysis of how infants processed the familiarization items, and a comparison of familiarization behaviour with novelty preference at test. To capture attention to commonalities we first calculated the mean proportion of leaf looking across the eight familiarization trials for each infant by dividing looking at the leaf by the sum of looking at shell and leaf (see above). Average leaf looking did not differ between the silent and labeling conditions (*M *=* *.33, *t*(56) = .27, *p *>* *.78, two‐tailed independent *t*‐test). However, infants in both conditions looked less at the leaf than the shell (Silent: *t*(28) = 9.36, *p *<* *.0001; Label: *t*(28) = 8.32, *p *<* *.0001). This is unsurprising, given the much lower variability of leaves compared to shells. Note that infants spent similar proportions of time looking at each part at the start of familiarization, as demonstrated by *t*‐tests against chance for trial 1 (Silent: *t*(28) = 1.63, *p *>* *.11; Label: *t*(25) = 1.05, *p *>* *.3; all two‐tailed). The low overall leaf‐looking proportions are therefore evidence for infants’ sensitivity to the greater variability (and therefore interestingness) of the shells.

We performed analyses of commonality preferences during familiarization analogous to those conducted by Althaus and Mareschal (2014), calculating the proportion of leaf looking for each 1000 ms timeslot. Unlike in Althaus and Mareschal's (2014) study these analyses did not reveal any effects of condition or block in the initial 1000 ms timeslot, and are therefore not reported here.^2^ We attribute this difference to the higher degree of similarity of common vs. variable parts in the current study.

### Relationship between familiarization and test performance

Next, we determined whether infants’ looking behaviour during familiarization correlated with their performance (i.e. novelty preference score) on test. In general, better encoding of a given object part during familiarization should lead to higher novelty preference on test when that part was inconsistent: Greater ‘sampling’ of leaves should lead to a better representation of leaves, making it easier to ‘reject’ an inconsistent leaf on test. The analogous relationship should hold for sampling of shells. Therefore, leaf looking during familiarization should be positively correlated with novelty preference on Test 1 (where Leaf_OCO_ is inconsistent with the familiarized set of leaves). By extension we would expect leaf looking during familiarization to be *negatively* correlated with performance on Test 2 (where Shell_OCO_ was inconsistent with the familiarized set of shells).^3^

**Figure 4:**
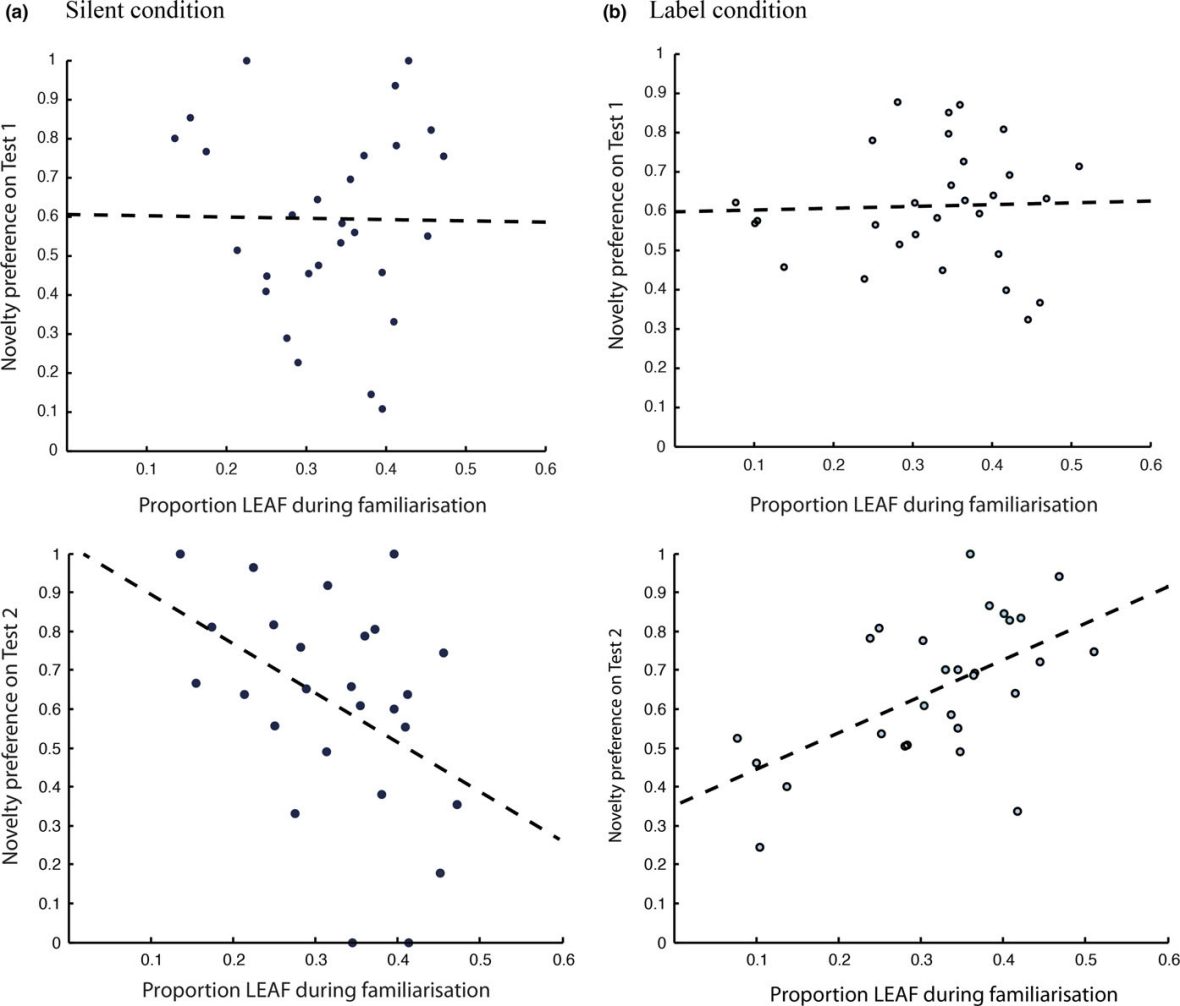
Relationship between familiarization and test performance for (a) silent condition (left panels) and (b) label condition (right panels). Top panels: relationship between familiarization and Test 1. Bottom panels: Relationship between familiarization and Test 2. The dashed lines represent the best linear fit.

In the silent condition, just one of the predictions was confirmed (see Figure 4(a)). There was no correlation between leaf looking during familiarization and novelty preference on Test 1 (*r *= −0.09, *p *>* *.66), but a significant *negative* correlation between leaf looking during familiarization and novelty preference on Test 2 (*r *= −0.45, *p *=* *.019). We interpret this asymmetry as reflecting the difference in variability between the object parts. Whereas the shells, which are highly variable, *require* extended sampling in order to capture the distribution of shapes (and recognize that Shell_OCO_ on Test 2 is inconsistent), the less variable leaves can be represented accurately with much less effort and sampling. As a consequence, Test 1 performance appears independent of familiarization behaviour.

Similarly, in the label condition (see Figure 4(b)), no significant relationship between leaf looking during familiarization and novelty preference on Test 1 was observed (*r *=* *.034, *p *>* *.86). However, the correlation between leaf looking during familiarization and novelty preference on Test 2 was *positive* (*r *=* *0.57, *p *<* *.002).^4^

Despite the apparent computational advantage of gaining a better knowledge of the variable feature's distribution through increased sampling, infants who focused more on the low‐variability feature (the leaf) during familiarization *with labels* achieved higher novelty preference scores on test. In contrast, infants who focused more on the leaf during familiarization *in silence* were less likely to show a novelty preference on test. These asymmetrical correlations of commonality focus (leaf looking) during familiarization and novelty preference at Test 2 suggest that labeling during familiarization has a distinctive impact on the process of visual category formation. We now explore this possibility further by examining infants’ attention to individual object parts at test.

### Part‐based looking during test

As demonstrated in the previous section, different processing trajectories lead to success when learning in silence and with labels. Infants exhibiting the highest amounts of object‐based novelty preference after learning are the ones who either learned in silence and focused on the highly variable feature, or who learned with labels and focused on the low‐variability feature. A critical question is therefore whether these differences in processing lead to an altered mental category representation. One hypothesis is that infants in the label condition have extracted the leaf as a commonality in the target category, and represent the category in a way that emphasizes this part. Analysing part‐based looking patterns on the test trials (as opposed to the object‐based novelty preference scores given above) can provide more insight into the validity of this hypothesis.

Recognition of the test objects as novel entails rejection of the relevant object part – Leaf_OCO_ in Test 1, and Shell_OCO_ in Test 2. However, do infants ‘reject’ the novel objects on the basis of this single part, or do they direct a substantial proportion of their looking at the remaining part, which itself should not appear novel? If infants in the label condition have learned a category representation that emphasizes the presence of the commonality, i.e. the familiar leaf, Test object 2 should be interesting (and therefore preferred) not just because it contains the novel part Shell_OCO_, but because it also contains the familiar‐looking leaf (Leaf_OCO_). We would therefore expect infants in the label condition to exhibit increased looking at Leaf_OCO_ on Test 2. By contrast, if the familiar leaf does not play a privileged role in its representation within the category, Leaf_OCO_ on Test 2 should not receive much looking as it is not novel at all. In particular, there should not be a difference in looking at Leaf_OCO_ between the label and silent conditions.

**Figure 5:**
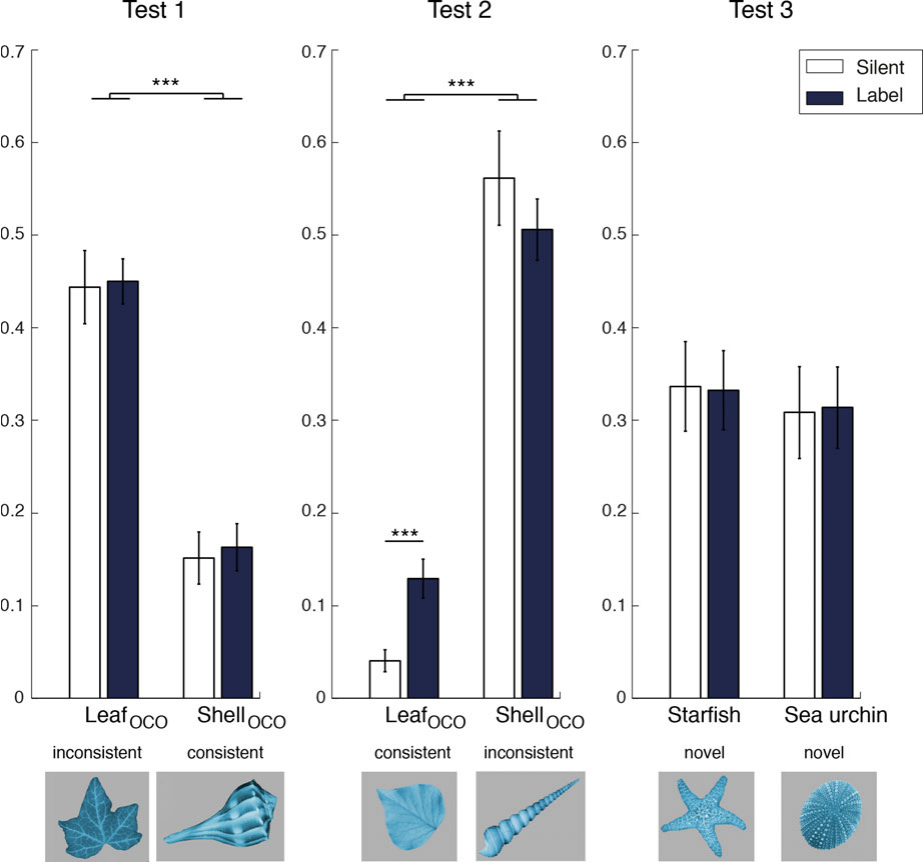
Part‐based looking during Tests 1, 2 and 3 (proportion of looking directed at each part, out of the four visible parts). Whiskers represent standard errors. Contrasts marked with *** are significant at the .001 level.

To address the question of part‐based looking at test, we calculated looking directed at each test object part separately for all test trials, as a proportion of the total time infants spent gazing at either object (i.e. any of the four parts Shell_OCO_, Leaf_OCO_, Shell_WCO_, Leaf_WCO_ on Tests 1 and 2, Starfish_OCO_, Sea‐urchin_OCO_, Shell_WCO_, Leaf_WCO_ on Test 3). Results are depicted in Figure 5. Infants in both conditions exhibited highly similar looking patterns on Test 3, confirming that both the starfish and sea urchin were recognized as novel and infants were clearly still engaged in the task. Infants in both the silent and labeling conditions treated Test object 1 similarly, with the majority of looking time being directed at Leaf_OCO_ (the inconsistent part; Silent: *M *=* *44.4%, *SE* = 4.0; Label: *M *=* *45.0%, *SE* = 2.4), and about 16% of looking spent gazing at Shell_OCO_ (Silent: *M *=* *15.2%, *SE* = 2.8; Label: *M *=* *16.3%, *SE* = 2.6). This level of attention to Shell_OCO_ is unsurprising given the overall variability of the shell during familiarization, which meant that even the shell contained in Test object 1 was relatively novel. For Test object 2 (which contained an inconsistent shell), infants in the silent condition all but ignored Leaf_OCO_ (*M *=* *4.1%, *SE* = 1.1). This is readily explained by the low variability of the leaves: at this stage the (consistent) leaf part should appear as highly familiar. However, infants in the label condition spent a considerable proportion of time looking at this highly familiar part (*M *=* *12.9%, *SE* = 2.1). Further analysis of Test 2 using a mixed‐level ANOVA with factors Part (Shell_OCO_, Leaf_OCO_) and Condition (Silent, Label) revealed a significant interaction of Part × Condition (*F*(1, 54) = 4.949, *p *=* *.03) and a main effect of Part (*F*(1, 54) = 191.2, *p *<* *.001). A planned comparison confirmed that infants in the label condition spent a higher proportion of time looking at Leaf_OCO_ compared to infants in the silent condition (*t*(54) = 3.6, *p *<* *.001, two‐sample *t*‐test, two‐tailed). Infants in the label condition clearly treated the Leaf_OCO_ – the commonality across exemplars – as a privileged object part, spending additional time gazing at it despite its lack of novelty.

### Relationship between part‐based looking during familiarization and test

The correlation between familiarization behavior and object‐based novelty preference at test in the label condition suggests that part‐based looking patterns at test should also exhibit a correlation with familiarization behavior and point to a causal relationship between familiarization and test behavior. Therefore, we next examine the relationship between the proportion of looking at Leaf_OCO_ in Test 2 and leaf looking during familiarization. In the label condition, infants who spent more time looking at the leaf during familiarization, also spent more time looking at Leaf_OCO_ in Test 2 (*r *=* *0.43, *p *=* *.024).^5^ In the silent condition, there is no such correlation (*r *= −.02, *p *>* *.93). However, there is a *negative* correlation in the silent condition between leaf looking during familiarization and Shell_OCO_ on Test 2 (*r *= −.46, *p *=* *.016), corresponding to the negative correlation between leaf looking during familiarization and object‐based novelty preference reported earlier for the silent condition. In other words, in the label condition, novelty preference on Test 2 is driven by looking at both the novel part and the attached familiar leaf, and this follows a leaf‐focus during familiarization. In the silent condition the opposite pattern is found. In this condition novelty preference on Test 2 is driven by looking at the shell, and follows a shell‐focus during familiarization.

### Vocabulary scores

CDI questionnaires were obtained for 53 of the infants, and production as well as comprehension scores were calculated. Neither comprehension scores (Silent condition: *M *=* *47, *SD* = 46; Label condition: *M *=* *71, *SD* = 63) nor production scores (Silent condition: *M *=* *3.6, *SD* = 6.2; Label condition: *M *=* *3.9, *SD* = 4.8) differed across conditions (Comprehension: *p *=* *.17, Production: *p *=* *.34, Wilcoxon rank‐sum). We also assessed whether there was a relationship between individual infants’ vocabulary scores (production or comprehension) and their performance on Test 1 or Test 2. After the removal of three outliers with production scores larger than two standard deviations above the mean the correlation between infants’ production scores and novelty preference on Test 2 in the silent condition was found to be far from significant (*r *=* *.228, *p *=* *.296). All other correlations between vocabulary scores and test performance were non‐significant (all *p*s* *> .38).

## Discussion

An important, though unsubstantiated, hypothesis is that labels facilitate infant visual categorization by *highlighting the commonalities between objects* (Waxman & Markow, 1995, p. 298). We have evaluated this claim by comparing infant performance on a visual categorization task in the presence of labels with the same task carried out in silence and monitoring infants’ attention to object parts during familiarization and test. We found weak evidence for a *facilitation* of categorization in the presence of labels. Infants’ novelty preference on Test trial 2 in the silent condition was only marginally significant and the majority of infants directed their gaze at the familiar object first – in contrast to infants in the label condition, whose first look was typically directed at the novel object. This indicates that the representation of the variable shell part was less robust than in the label condition. On the whole, however, infants in both conditions show evidence of successful categorization (see Table 1).

Contrasting the similar overall categorization performance we find evidence that labels impact the *manner* in which infants process a novel object category and that labels encourage infants to focus on the common parts of objects in the category. Three outcomes support this interpretation:

1. Labels help maintain attention to objects during familiarization compared to object familiarization in silence (see Figure 3).
2. Infants who focused more on the common part (the leaf) during familiarization when objects were labeled were more likely to show a novelty preference at test. In contrast, infants who focused more on the common parts when familiarized in silence were less likely to show a novelty preference at test (see Figure 4).
3. Infants familiarized with labels paid more attention to the common part of the out‐of‐category object at test than infants familiarized in silence (see Figure 5).

Note that infant performance at Test 2 is critical for evaluating the *manner* in which labels impact category formation. In Test 1, the out‐of‐category object is highlighted by the novelty of the leaf component. Sensitivity to this variation is apparent to infants in both conditions, as would be expected if infants were responding to the degree of deviation from the familiarization stimuli. However, the lack of variability of the leaf component in Test 2 should *not* encourage differential levels of attention across the two conditions, if infants are merely responding to novelty. Nevertheless, infants paid more attention to this common part in Test 2 in the label condition than the silent condition, suggesting that they had assigned the leaf a special status. Furthermore, infants who focused more on the leaf component during familiarization in the label condition continued to do so at Test 2. This correlation did not hold for the silent condition. Taken together, these results indicate that, in the silent condition, infants are governed exclusively by the variability of object parts. In the label condition, infants are more inclined to attend to shared category features.

We interpret these looking patterns as a difference in feature‐weighting. While infants in the silent condition learn about both the leaf and shell parts, as demonstrated by their novelty preferences at test, they do not seem to attribute a specific role to the leaves as a recurring element. By contrast, the leaves appear to be particularly important for infants in the label condition – as if they assign a greater weight to this object part in the process of accepting or rejecting an object as a part of the target category.

It is possible to explain this behavior as the outcome of signal co‐occurrence. During familiarization with labels, infants repeatedly heard the label ‘timbo’ together with varying visual stimuli whose shared attribute is that they possess a specific type of leaf. When associating auditory and visual stimuli, therefore, the strongest association should occur between the label and the leaf. The leaf therefore takes on the quality of a *diagnostic* feature for the category ‘timbo’. While a feature can theoretically only be considered truly diagnostic if it can be established that it occurs in no other context (which is impossible in the single‐category scenario used here), the recurring nature of this feature can still be picked up by the cognitive system as indicative of category membership. On test, when infants are confronted with an object in the absence of a label, and this object further does *not* conform to the expected feature distribution (due to the novelty of the shell part), the leaf can be used as an indicator that the observed object is indeed a valid category member (for instance, on encountering a new breed of dog hearing it bark may confirm that this is indeed just an odd member of an already familiar category). What we observe on Test 2 in the Label condition is, then, the combination of two effects: (a) responding to the novelty of the out‐of‐category shell, and (b) responding to the label‐induced diagnosticity of the familiar leaf.

The objects used in this study were constructed to consist of spatially separate object parts in order to use gaze patterns to tap into feature processing. Many natural categories do not possess such localized features. While eye movements cannot be used to assess feature extraction in these cases (imagine, for instance, in categorizing land animals vs. sea animals), it seems plausible that the underlying computational processes which here give rise to fixations directed towards the common object part are nevertheless the same.

In this experiment, infants in both conditions successfully formed a target category. The result is therefore different in nature from the majority of studies focusing on the impact of labels on categorization. Typically, studies report category formation by infants in the presence of labeling events but not in their absence (e.g. Fulkerson & Waxman, 2007; Waxman & Markow, 1995), or they involve a category that is learnable in silence but where infants fail to categorize in the presence of a novel label (e.g. Robinson & Sloutsky, 2007). In other words, the visual categories used to familiarize infants differ a priori with regard to whether infants will or will not learn them without additional information. It is therefore hard to evaluate *how* labels influenced categorization in each of these cases. While our results do not address the question of whether labels facilitate or disrupt learning, they shed light on how object processing changes when labels are provided. Learning occurs in both conditions, but what is learned depends on whether or not labels were heard. The results confirm the hypothesis that labels direct infants’ attention to commonalities during category formation. While the *mechanism* underlying this behavior is most likely a (partial) cross‐modal matching process, the outcome is the identification of parts with an increased weighting for similarity assessment – a step on the way to forming a diagnostic criterion. Clearly, labels are powerful tools that highlight the relevance of individual features for object identification and categorization.

## Notes

1 Since some infants did not contribute looking to *all three* test trials, the mixed effects ANOVA only includes a subset of *N* = 55 infants. The planned comparisons for each individual test trial include all infants for whom looking was recorded on the relevant trial. In the silent condition, two infants failed to look at the screen (no gaze recorded) on Test 2, and three failed to look at the screen during Test 3.

2 With the exception of Slot 4 (3000–4000 ms after trial onset), where there was a significant Block by Condition interaction (*F*(1, 53) = 4.32, *p *=* *.043), no significant effects were found (all *F*s < 2.49, *p*s* *> .12). Considering that there was no specific hypothesis for Slot 4, we do not believe that the significant interaction in this slot reflects a cognitive effect.

3 Recall that the proportion of shell looking (p_shell_) during familiarization is just the proportion of time remaining when the infant is not looking at the leaf, so p_shell_ = 1 − p_leaf_. Novelty preference on Test 2 is a measure of sensitivity to shell variability. Detection of shell variability is likely to be enhanced by longer looking at the shells during familiarization. Hence, p_leaf_ should be negatively correlated with any novelty preference in Test 2.

4 This result was obtained after exclusion of a single outlier identified after obtaining the best linear fit. The excluded datapoint was the only one further from the line than two standard deviations of the residuals. Including this datapoint the correlation coefficient was *r *=* *0.37, *p *=* *.048.

5 This result was obtained after exclusion of the same single outlier excluded previously. Including this data point the correlation coefficient was *r *=* *.36, *p *=* *.057.
